# Oligouridylate Binding Protein 1b Plays an Integral Role in Plant Heat Stress Tolerance

**DOI:** 10.3389/fpls.2016.00853

**Published:** 2016-06-17

**Authors:** Cam Chau Nguyen, Kentaro Nakaminami, Akihiro Matsui, Shuhei Kobayashi, Yukio Kurihara, Kiminori Toyooka, Maho Tanaka, Motoaki Seki

**Affiliations:** ^1^Plant Genomic Network Research Team, RIKEN Center for Sustainable Resource ScienceYokohama, Japan; ^2^Kihara Institute for Biological Research, Yokohama City UniversityYokohama, Japan; ^3^Synthetic Genomics Research Group, Biomass Engineering Program Cooperation Division, RIKEN Center for Sustainable Resource ScienceYokohama, Japan; ^4^Mass Spectrometry and Microscopy Unit, RIKEN Center for Sustainable Resource ScienceYokohama, Japan; ^5^Core Research for Evolutional Science and Technology, Japan Science and TechnologyKawaguchi, Japan

**Keywords:** UBP1b, UBP1b stress granule, heat stress tolerance, RNA stability

## Abstract

Stress granules (SGs), which are formed in the plant cytoplasm under stress conditions, are transient dynamic sites (particles) for mRNA storage. SGs are actively involved in protecting mRNAs from degradation. Oligouridylate binding protein 1b (UBP1b) is a component of SGs. The formation of microscopically visible cytoplasmic foci, referred to as UBP1b SG, was induced by heat treatment in *UBP1b*-overexpressing *Arabidopsis* plants (*UBP1b*-ox). A detailed understanding of the function of UBP1b, however, is still not clear. *UBP1b*-ox plants displayed increased heat tolerance, relative to control plants, while *ubp1b* mutants were more sensitive to heat stress than control plants. Microarray analysis identified 117 genes whose expression was heat-inducible and higher in the *UBP1b*-ox plants. RNA decay analysis was performed using cordycepin, a transcriptional inhibitor. In order to determine if those genes serve as targets of UBP1b, the rate of RNA degradation of a DnaJ heat shock protein and a stress-associated protein (AtSAP3) in *UBP1b*-ox plants was slower than in control plants; indicating that the mRNAs of these genes were protected within the UBP1b SG granule. Collectively, these data demonstrate that UBP1b plays an integral role in heat stress tolerance in plants.

## Introduction

Plants are strongly affected in a negative manner by adverse environmental stress conditions, such as high temperature, cold weather, drought, and high salinity. The regulation of mRNA, including mRNA degradation and stabilization, is one of the mechanisms used by plants to effectively adapt to abiotic stress (Nakaminami et al., 2012). Several types of particles exist in the cytoplasm of plants, such as stress granules (SGs; Kedersha et al., 1999) and processing bodies (P-bodies), that contribute to mRNA regulation (Bashkirov et al., 1997; Gibbings et al., 2009). P-bodies are involved in mRNA degradation, while SGs are involved in mRNA stabilization.

SGs are cytoplasmic particles comprised of proteins and RNAs. Previous reports indicated that translation initiation factors, the 40S ribosomal subunit, poly(A)-binding protein, and some other RNA-binding proteins were all found within SGs (Nover et al., 1989; Kedersha et al., 1999, 2002). SGs form in the cytosol when cells are exposed to stress conditions (Nover et al., 1983; Kayali et al., 2005). A major function of SG is to protect RNAs from adverse conditions within the cell that result from severe environmental stress; thus the presence of SGs is highly correlated to stress conditions (Nover et al., 1989). SGs mediate post-transcriptional gene regulation. They are comprised of various structures and components and their overall composition is dependent upon the specific stress condition to which the plant is exposed (Ivanov and Nadezhdina, 2006; Buchan et al., 2011). UBP1b (oligouridylate binding protein 1b) is a known protein component of SGs that form in response to heat stress (Lambermon et al., 2000; Weber et al., 2008).

Most mRNAs are degraded under heat stress conditions unless they are protected in some manner during post-transcriptional processes. UBP1b, a known component of SGs, has been reported to function in protecting mRNAs from degradation. UBP1b has three RNA-binding domains (RBDs) that recognize mRNA 3′-UTRs, U-rich introns, and poly (A) tails. When UBP1b interacts with the 3′-UTR of mRNAs, it protects them from degradation. UBP1b is localized in both nuclei and SGs (Lambermon et al., 2000; Weber et al., 2008). The function of UBP1 in the response to various stresses has been previously studied. McCue et al. (2012) reported that *ubp1b* mutants are sensitive to high-salinity and osmotic stress conditions. In addition, they also demonstrated that stress sensitivity may be epigenetically regulated by the transposable element (TE)-derived siRNA854 that targets the *UBP1b* 3′-UTR (McCue et al., 2012). However, details pertaining to the molecular mechanisms which function to regulate stress-sensitivity, such as the identity of UBP1b-targeted mRNAs, have not been well documented. Sorenson and Bailey-Serres (2014) reported that *ubp1c* mutants are hypersensitive to hypoxia stress and exhibit a sucrose-repressible post-germination arrest phenotype (Sorenson and Bailey-Serres, 2014). The use of a messenger ribonucleoprotein (mRNP) immunoprecipitation assay indicated that UBP1C, a homolog of UBP1B, associates with the uracil-rich 3′-untranslated regions (UTRs) of mRNAs (Sorenson and Bailey-Serres, 2014).

It is important to not only understand the mechanism by which UBP1b protects mRNAs from degradation but also to identify the target mRNAs that interact with UBP1b. Understanding the function of UBP1b in plants would increase our knowledge of how plants respond to environmental stress. In the present study, a functional analysis of UBP1b in abiotic stress response was conducted in order to better understand the function of UBP1b SG.

Confocal Laser Scanning Microscopy (CLSM) was used to observe cells of *UBP1b-Venus*-overexpressing (ox) transgenic *Arabidopsis* plants subjected to different abiotic stress conditions in order to determine the specific stress conditions that induce UBP1b SG formation. The Venus reporter protein, which is an improved version of YFP, was utilized in a previously reported study (Nagai et al., 2002). The localization of UBP1b was found to change in response to heat stress. In addition, it was determined that the formation of new UBP1b SGs is induced by heat stress. These data indicated that UBP1b may play an important role in plant response to heat stress. The study further demonstrated that *UBP1b*-ox plants have a higher level of heat stress tolerance than control plants. Lastly, the study also identified several candidate target mRNAs of UBP1b that may function in heat stress response.

## Materials and methods

### Plant material and growth conditions

All experiments conducted in the present study utilized 35S::*Venus-UBP1b*-overexpressing (*UBP1b*-ox) *A. thaliana* plants (ecotype: Columbia) and 35S::*Venus* (Venus) *Arabidopsis* plants (ecotype: Columbia) as a control.

Transgenic *Arabidopsis* plants were produced as follows. The full-length *UBP1b* fragment was cloned from a cDNA library using the primers UBP1bf: 5′-GGGGTACCGGAAAATGGGTAGCAAGATG-3′ and Ubp1br: 5′-CGAGCTCAGGGTTTAAGCTTGGCTTCC-3′.

The *UBP1b* fragment was subsequently fused with *Venus* (Nagai et al., 2002) and introduced into the pYY45 vector, which contains a 35S promoter, to create a 35S::*Venus*-*UBP1b* construct. A 35S::*Venus* construct was used as a control. The constructs were introduced into *Arabidopsis* plants using *Agrobacterium tumefaciens* (strain GV 3101) and the floral-dip method (Clough and Bent, 1998). The plants were grown on Murashige and Skoog (MS) agar medium under long day conditions (16 h light/8 h dark) at 22°C in an environmental chamber (TOMY CF-405, Tokyo, Japan) and used in all of the subsequent experiments.

Two lines of *ubp1b* mutants, mutant 1 and 2 (FLAG_071F09 and FLAG_298B04, WS background), were obtained from *Arabidopsis* Biological Resource Center (ABRC) and used in the heat tolerance assay. Wild-type *Arabidopsis* plants (ecotype: WS) were used as a control. All of the plants were grown under the same condition as *UBP1b*-overexpressing plants.

### Microscopy

Leaves and roots of 14-day-old *Venus-UBP1b*-overexpressing (*UBP1b*-ox) and 35S::*Venus* (*Venus* control) plants were examined under a Zeiss CLSM 700, Confocal Laser Scanning Microscope (ZEISS, Oberkochen, Germany). Images of non-treated (22°C), heat-treated (40°C and 37°C for 1 h), or recovery after heat-treated (returned back to 22°C for 3, 6, and 12 h after 1 h of 40°C treatment) samples mounted in water were obtained using a 40 × Plan-Apochromat lens. A diode laser with 488 nm excitation and filters (488–555 nm, 560–700 nm) were used to observe the fluorescence of Venus and autofluorescence of chloroplasts. Data were analyzed by ZEN 2011 software (ZEISS, Oberkochen, Germany).

### Heat-stress tolerance assay

Heat-stress tolerance assays were conducted using 14-day-old *UBP1b*-ox (ecotype: Columbia) and *ubp1b* mutant (ecotype: WS) plants. Twelve plants were subjected to 42°C for 3 h in an incubator (Panasonic, Kadoma, Japan) after which they were returned to 22°C. The number of surviving plants were counted at 14 days after the heat treatment and the percentage of survival was calculated based on 3 biological replicates of sixty plants.

### Microarray analysis

Fourteen-day-old *UBP1b*-ox and *Venus* control plants were either subjected to 40°C for 1h (treated) or left at 22°C (non-treated). Total RNA was extracted from whole seedlings using the Plant RNA Reagent (Thermo Fisher Scientific, Waltham, MA, USA). Three biological replicates were used, where each biological replicate consisted of a pool of five seedlings. RNAs were reverse transcribed into cDNAs using 400 ng of total RNA. cDNA was labeled with a single color (Cy3) using a Quick Amp labeling kit (Agilent Technologies, Palo Alto, CA, USA) and hybridized to *Arabidopsis* custom microarrays (Nguyen et al., 2015) (GEO array platform: GPL19830, Agilent Technologies). Arrays were scanned with a microarray scanner (G2505B, Agilent Technologies). The resulting microarray data were deposited in and are available on the GEO website (GEO ID: GSE78713).

Microarray data for treated and non-treated plants was obtained from three biological replicates. The fluorescence intensities of the microarray probes were normalized by quantile normalization, using the limma package (Smyth, 2004). The intensity data from multiple samples were compared using a controlled p-value (FDR) and a one-way analysis of variance (ANOVA) with a *p* value of < 0.0001 in order to control the level of false positives obtained between the samples and treatments. Genes with a significant change in expression were selected using the following criteria: an expression log_2_ ratio > 0.7 and a *p*-value of the Student's *t*-test < 0.15, relative to the control, as a *post-hoc* test. The R program ver. 2.12.1 was used for the analysis of the microarray data.

### RT-qPCR

Total RNAs were prepared from samples (3 biological replicates) using the Plant RNA Reagent (Thermo Fisher Scientific) and cDNAs were synthesized using a Quantitech cDNA synthesis kit (Qiagen, Venlo, Netherlands). The cDNAs were subsequently used as templates for RT-qPCR analyses that were performed using Fast SYBR Green MasterMix (Thermo Fisher Scientific) and a StepOne Plus Real Time PCR system (Thermo Fisher Scientific). The *YLS8* gene was used as a reference gene for the normalization of the expression data. Primers used in the RT-qPCR analyses are listed in Table S1. RT-qPCR data were analyzed using StepOne Plus software (Thermo Fisher Scientific).

### RNA decay analysis

Cordycepin was used as a transcription inhibitor in the RNA decay assay. Twenty of 2-week-old plants were treated with 10 ml of water containing 0.6 mM of cordycepin at 22°C for 2 h and five seedlings of plant samples were collected every 30 min (3 biological repeats were performed on a pool of 75 plant samples). Total RNA was prepared from cordycepin-treated samples and cDNA was synthesized as described above. RT-qPCR was conducted in order to determine the level of mRNA. Decay rates were calculated based on the log2 value of the relative expression of the target genes. *RIDA (AT3G20390), SUMO2 (AT5G55160)*, and *AT2G23090*, whose transcripts have been reported to have a half-life longer than 24 h, were used as negative controls (Narsai et al., 2007). Primers used in the RT-qPCR analysis are listed in Table S1.

## Results

### Heat stress induces the formation of UBP1b stress granules

**Figure 1:**
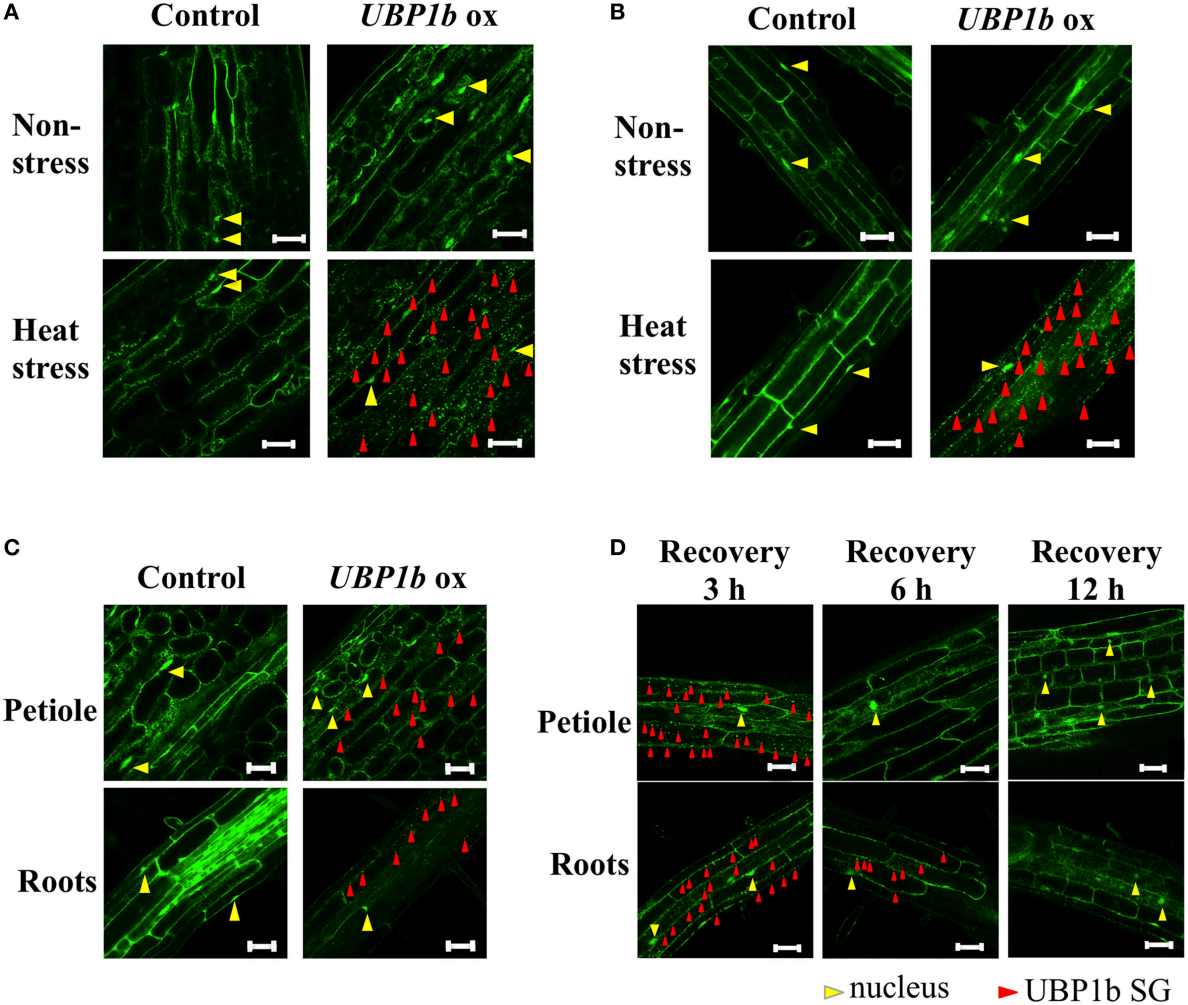
**Subcellular localization of UBP1b in plants under normal and heat stress conditions**. Red and yellow triangles identify the presence of UBP1b SGs and nuclei, respectively. Scale bar = 50 μm. **(A)** Localization of UBP1b in the cells of petioles of 2-week-old *UBP1b*-ox and *Venus* control plants exposed to non-stress (22°C) and heat stress (40°C for 1 h). **(B)** Subcellular localization of UBP1b in the root cells of UBP1b-ox and *Venus* control plants exposed to non-stress (22°C) and heat stress (40°C for 1 h). **(C)** Subcellular localization of UBP1b in *UBP1b*-ox and *Venus* control plants subjected to a mild heat stress (37°C for 1 h) prior to observation. **(D)** Subcellular localization of UBP1b in petiole and root cells of *UBP1b*-ox plants after recovery from heat stress. After exposure to 40°C for 1 h, plants were returned to 22°C and maintained for an additional 3, 6, or 12 h prior to observation.

Previous observations indicated that SGs are formed in response to heat stress and that UBP1b is localized within the SGs (Weber et al., 2008). In order to confirm these results, the *UBP1b*-ox and *Venus* control plants were subjected to a heat stress and subsequently observed using confocal laser scanning microscopy (CLSM). The expression of *UBP1b* gene was higher in *UBP1b*-ox lines compared to *Venus* control plants (Figure S1A). Under normal (22°C) conditions, the signals were localized in the nuclei of petiole cells in both *UBP1b*-ox and *Venus* control plants (Figure 1A, upper panels). In response to heat stress (1 h at 40°C), UBP1b in *UBP1b*-ox plants (2 lines: ox 1 and ox 2) became localized and visible in cytoplasmic foci, referred to as UBP1b SGs (Figure 1A, lower right panel). In contrast, there was little change in the localization of the signals in control plants and it was similar to what was observed under normal (22°C) conditions (Figure 1A, lower left panel). Similar results were observed in root cells of *Arabidopsis* (Figure 1B). In relative comparison to the intensity of the signals observed when *UBP1b*-ox plants were subjected to a severe heat stress (40°C), these cytoplasmic foci of UBP1b signals in the *UBP1b*-ox plants were weaker and less abundant when plants were subjected to a mild heat stress (37°C) (Figure 1C). The intensity of the signals in the cytoplasm was reduced after plants were allowed to recover at 22°C for 3 h (Figure 1D). After 6 h of recovery, the signals totally disappeared from the cytoplasm within petiole cells. On the other hand, a few dots of signal were still observed in the cytoplasm of root cells (Figure 1D). After 12 h of recovery, all of the signals disappeared from the cytosol of both petiole and root cells (Figure 1D). Based on these observations, it was concluded that heat stress induces UBP1b SG formation.

### UBP1b is involved in heat stress tolerance

**Figure 2:**
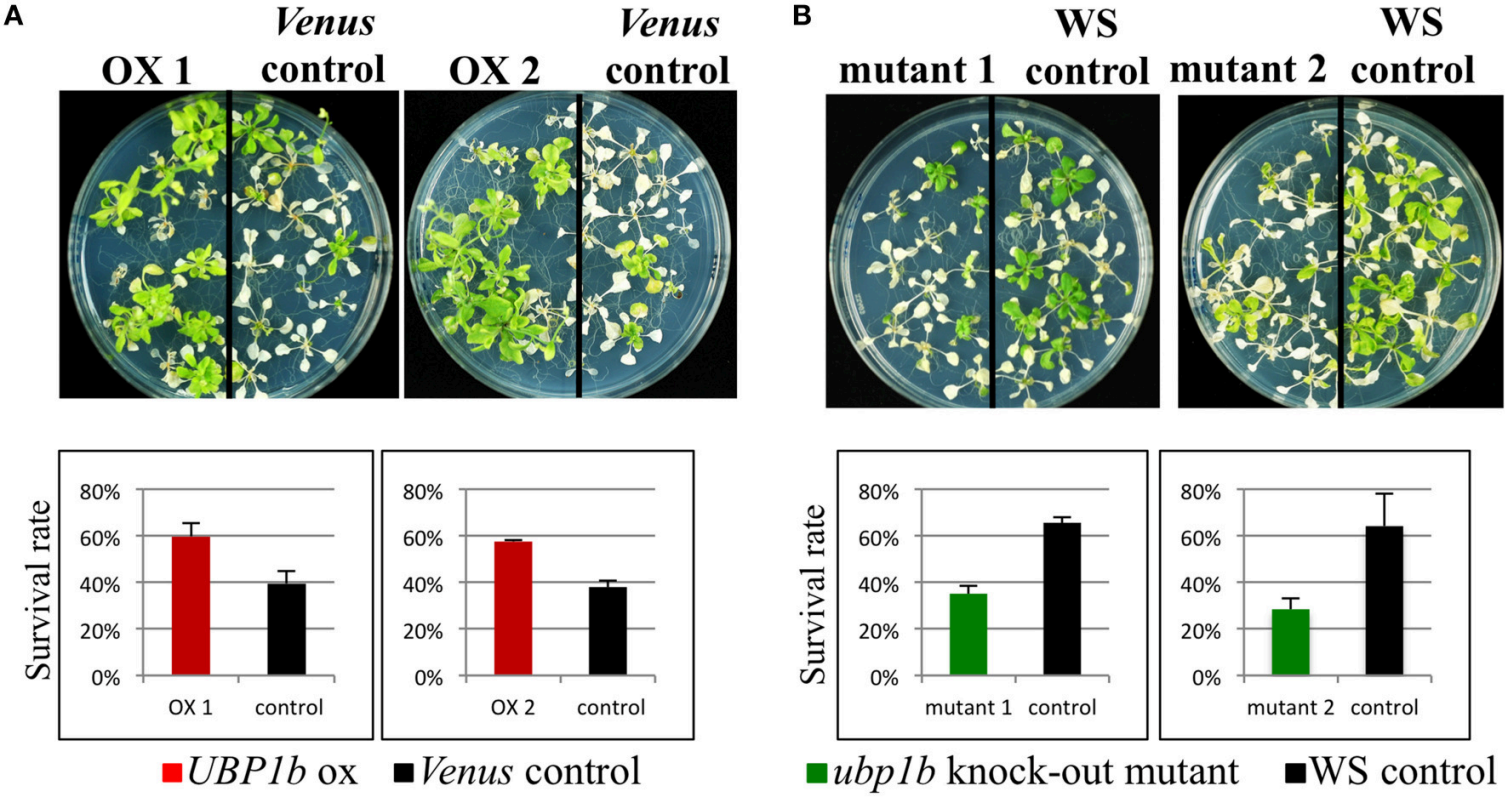
**Heat tolerance assay**. Two-week-old *UBP1b*-ox (2 lines: ox 1 and ox 2) and *Venus* control plants were subjected to 42° C for 3 h, and then grown at 22° C under long-days for 2 weeks prior to assessing plant survival. Twelve seedlings of each line were sown on each plate, and five plates of plants were used in each assay. The assay included three biological replicates. **(A)** Phenotype and survival rate of *UBP1b*-ox and *Venu*s control plants at the termination of the heat stress tolerance assay. **(B)** Phenotype and survival rate of *ubp1b* mutants and *WS* control plants at the termination of the heat stress tolerance assay. Y-axis represents the percentage of surviving plants relative to the total number of plants used within each assay. Data represent the mean ± sd of three biological replicates.

Although the formation of UBP1b SG in response to heat stress was confirmed, the effect of *UBP1b* overexpression on the phenotype of plants exposed to heat stress has not been documented. Therefore, a heat stress tolerance assay was conducted using *UBP1b*-ox and *Venus* control plants. Plants were subjected to 42°C for 3 h (Figure 2A). Two lines of *UBP1b*-ox plants exhibited a significantly higher rate of survival than the *Venus* control plants. Additionally, two *ubp1b* mutants subjected to the same heat stress were more sensitive (lower rate of survival) to the heat stress than wild type plants (Figure 2B). The expression of *UBP1b* gene was not detected in the *ubp1b* mutants (Figure S1B). Since two different ecotype backgrounds were used in this experiment, the heat sensitivity of WT differed between Columbia and WS ecotypes. These results indicate that UBP1b is somehow involved in the adaptation of plants to heat stress.

### Microarray analysis and the identification of candidate target genes of UBP1b associated with heat-stress response

**Figure 3:**
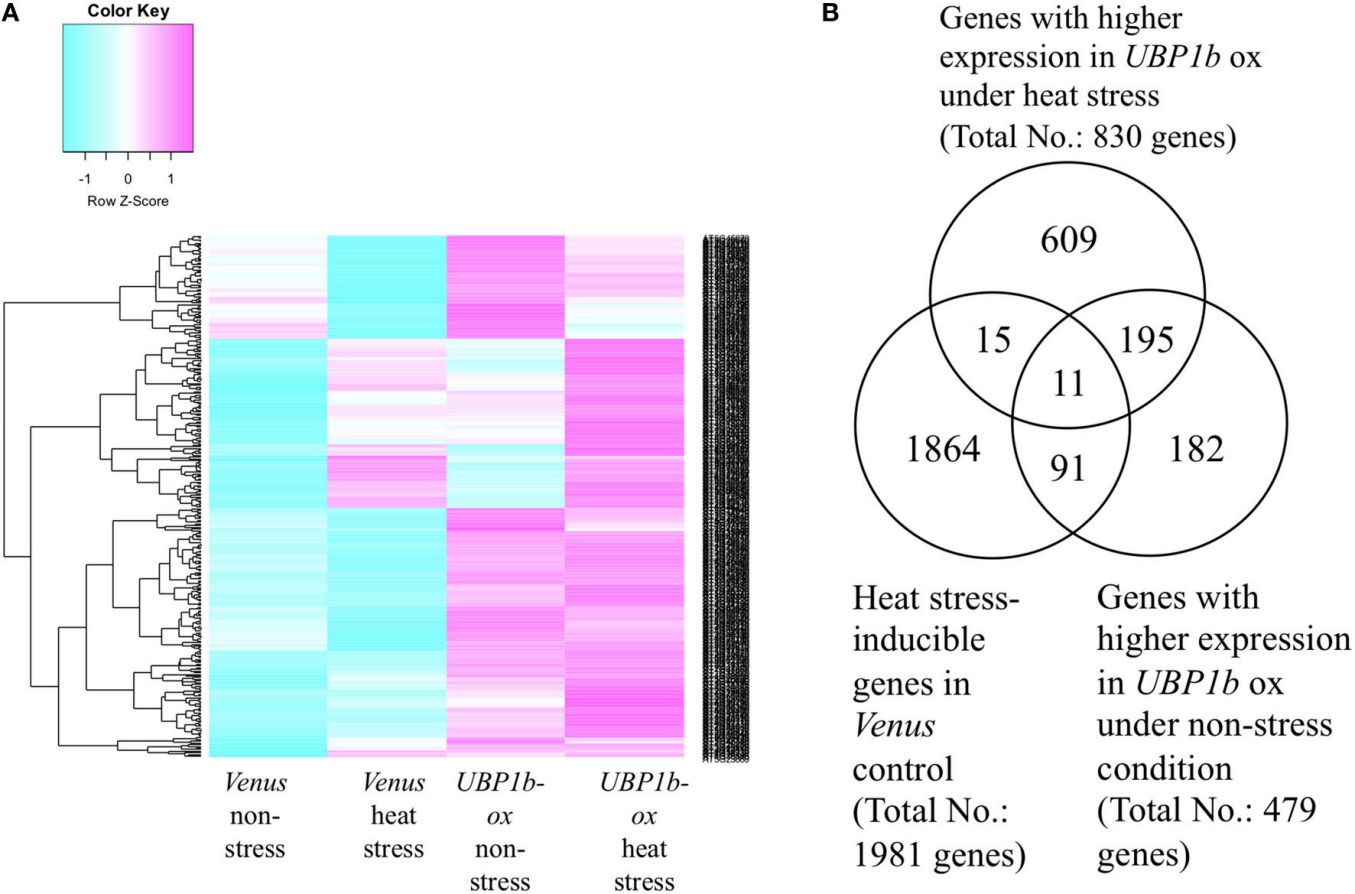
**Microarray analysis of ***UBP1b***-ox and ***Venu***s control plants subjected to heat stress and non-stress conditions**. Two-week-old *Arabidopsis* plants were subjected to 40°C for 1 h. Each sample was composed of five pooled seedlings which were used for extracting total RNA. Three biological replicates were analyzed. **(A)** Heat map of the microarray expression data obtained for *UBP1b*-ox (line ox1) and *Venu*s control plants. Blue color refers to a negative z-score; pink color refers to a positive z-score of the level of gene expression. **(B)** Venn diagram representation of 1981 heat-inducible genes identified in the microarray analysis. A total of 479 genes exhibited higher levels of expression in *UBP1b*-ox plants than in *Venu*s control plants when subjected to non-stress conditions, and 830 genes exhibited higher levels of expression in *UBP1b*-ox plants than in *Venu*s control plants when subjected to heat-stress.

A microarray analysis was conducted using *UBP1b*-ox and *Venus* control plants subjected to non-stress (22°C) and heat-stress (40°C) conditions for 1 h. Four hundred and seventy-nine genes exhibited higher levels of expression in the *UBP1b*-ox plants, relative to *Venus* control plants in the non-stress condition (*p* < 0.15, FDR < 0.0001) (Figure 3, Table S2). Among these differentially expressed genes, 102 of them were further up-regulated by heat stress (Table S3). A total of 830 genes exhibited higher expression in *UBP1b*-ox plants exposed to heat stress, relative to *Venus* control plants exposed to heat stress (Figure 3, Table S4). Among those 830 genes, the expression of 26 genes was up-regulated by heat stress in *Venus* control plants. (Figure 3, Table S5). The expression of 1981 genes in *Venus* control plants was induced by the heat stress (Table S6). There was a total of 206 genes in the *UBP1b-ox* whose expression level was higher, relative to the *Venus* control plants, under both non-stress and heat stress conditions (Figure 3, Table S7). Among them, 11 genes were heat-inducible (Table S8).

Among the genes that were up-regulated by heat stress and whose expression was higher in the *UBP1b*-ox plants than in *Venus* control plants, a number of genes were identified whose functions are related to heat-stress response and tolerance (Figure 3, Table 1, Tables S3, S5). These included: (1) three DnaJ domain containing proteins (*AT1G56300, AT1G72416, AT3G13310*) which function in maintaining protein homeostasis under environmental stress conditions by stimulating the ATPase activity of chaperone proteins, such as 70-kilodalton heat shock proteins (Hsp70s) (Rajan and D'silva, 2009; Chiu et al., 2013); (2) a stress-associated protein (*AtSAP3*)/*AT2g27580* characterized as an A20/AN1-like zinc finger family protein (Kim et al., 2015); (3) two heat shock transcription factors (*HSFA4A/AT4G18880*, and *HSFA3/AT5G03720*) (Pérez-Salamó et al., 2014); (4) a succinic semialdehyde dehydrogenase (*SSADH, AT1G79440*) (Bouché et al., 2003); and (5) *WRKY25* (*AT2G30250*) whose overexpression was reported to enhance heat stress tolerance (Li et al., 2009).

**Table 1:** **List of heat stress response-related genes whose expression was higher in ***UBP1b***-ox plants than in ***Venus*** control plants**.

**AGI code**	**Gene name/Encoded protein**	**Non-stress**		**Heat stress**		**Ratio (ox/*Venus*) under non-stress^b^**	**Ratio (ox/*Venus*) under heat stress^c^**	**Ratio (heat/*non-stress*) in *Venus*^d^**	**Ratio (heat/*non-stress*) in *ox*^e^**
		***Venus* control^a^**	***UBP1b*-ox^a^**	***Venus* control^a^**	***UBP1b*-ox^a^**				
AT1G56300	DnaJ	7.2	9.5	10.7	11.5	2.3	0.8	3.6	2.0
AT1G72416	DnaJ	5.9	7.5	11.4	12.2	1.6	0.8	5.5	4.7
AT3G13310	DnaJ	12.3	13.6	12.8	14.6	0.5	0.9	1.4	1.8
AT4G18880	HSFA4A	8.8	9.9	9.9	11.2	1.0	1.3	1.0	1.3
AT5G03720	HSFA3	6.1	7.2	9.2	10.0	1.1	0.8	3.1	2.8
AT2G27580	AtSAP3	8.3	11.0	9.6	12.8	1.4	1.8	2.7	3.2
AT1G79440	SSADH	8.4	9.3	9.4	9.8	0.9	0.4	0.9	0.5
AT2G30250	WRKY25	8.5	9.1	9.6	11.1	1.2	1.9	0.7	1.4

a *Average of signal intensity in 3 biological replicates*.

b *Average of the log_2_ ratio of normalized signal value in UBP1b-ox vs. Venus control under non-stress condition (p < 0.15; FDR < 0.0001)*.

c *Average of the log_2_ ratio of normalized signal value in UBP1b-ox vs. Venus control under the heat stress condition (p < 0.15, FDR < 0.0001)*.

d *Average of the log_2_ ratio of normalized signal value in Venus control in the heat stress condition vs. non-stress condition (p < 0.15, FDR < 0.0001)*.

e *Average of the log_2_ ratio of normalized signal value in UBP1b-ox in the heat stress condition vs. non-stress condition (p < 0.15, FDR < 0.0001)*.

**Figure 4:**
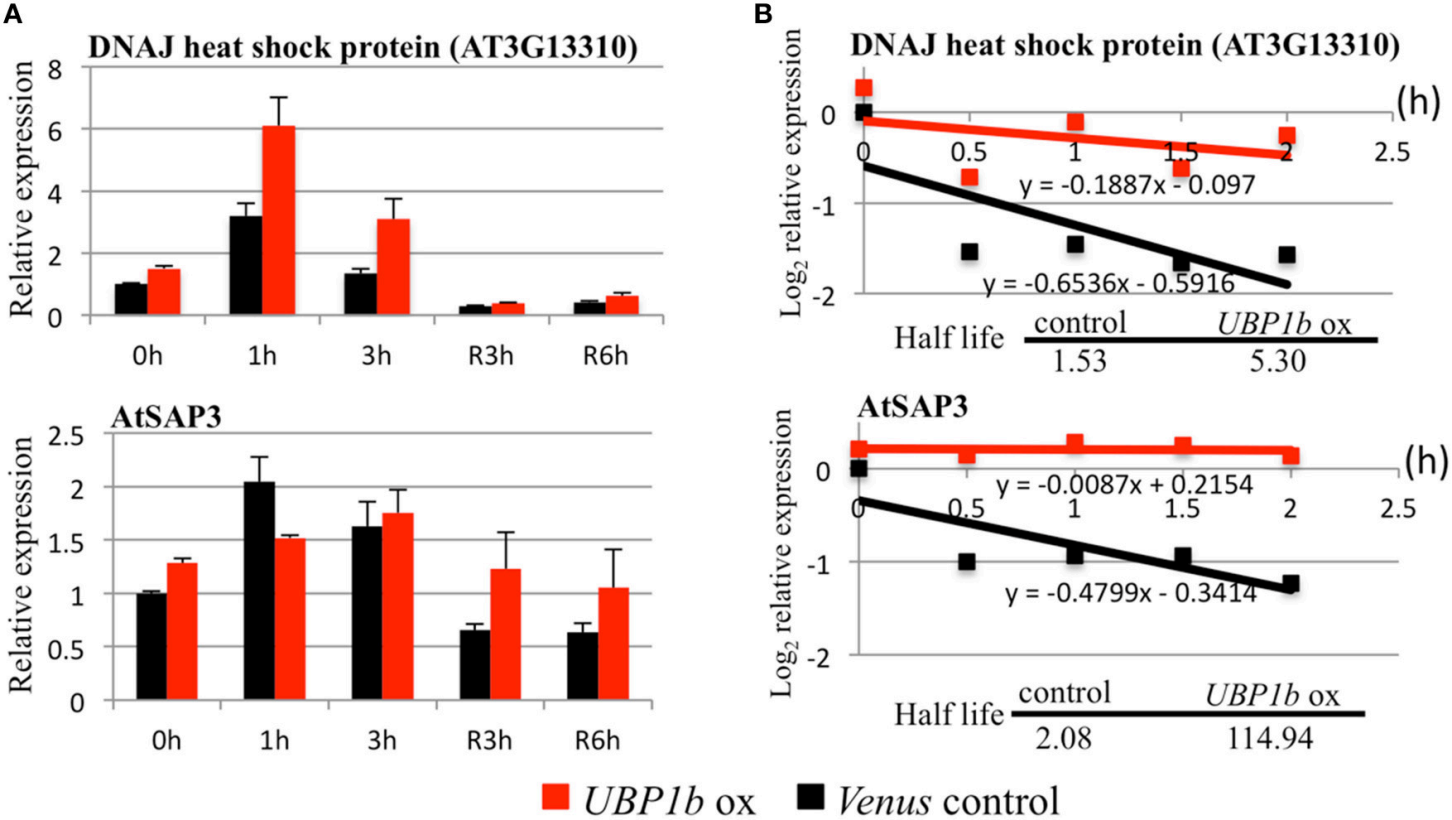
**RT-qPCR and mRNA decay analyses of UBP1b target genes**. Two UBP1b-targeted mRNAs (a DnaJ heat shock protein, AT3G13310 and a stress-associated protein, AtSAP3) were analyzed in *UBP1b*-ox (line ox1) and *Venus* control plants were analyzed. **(A)** RT-qPCR analysis of UBP1b target genes. Fourteen day-old plants were treated with heat (40°C) for 1, 3 h and then allowed to recover at 22°C for 3 h (R3h) and 6 h (R6h). Total mRNAs were extracted for RT-qPCR analysis. X-axis: time of treatment. Y-axis: relative expression of target mRNAs. Data represent the mean ± sd of three biological replicates. **(B)** mRNA decay analysis of UBP1b target genes.

In order to confirm the effect on the expression level of the aforementioned genes that were altered by *UBP1b* overexpression and heat treatment, RT-qPCR was performed using the same samples that were evaluated with microarray analysis (Figure 4, Figure S2). Under control and/or heat treated conditions, gene expression of the three DnaJ domain-containing proteins (*AT3G13310, AT1G72416*, and *AT1G56300*), *AtSAP3*, two heat shock transcription factors (*HSFA4A* and *HSFA3*), *SSADH*, and *WRKY25* were all higher in *UBP1b*-ox plants than in *Venus* control plants (Figure 4A, Figure S2). RT-qPCR analysis also confirmed that the expression of the three DnaJ domain-containing proteins (*AT3G13310, AT1G72416* and *AT1G56300*), *AtSAP3, SSADH*, and the two heat shock transcription factors (*HSFA4A* and *HSFA3*) was heat stress-inducible (Figure S2). The expression of *WRKY25* was not heat-inducible (Figure S2).

### RNA decay analysis

Although several candidate target mRNAs of UBP1b were identified, the mechanism by which UBP1b, as well as SGs, affect those targets mRNAs are not well understood. Based on the ability of UBP1b to bind to the 3′-UTR domain of mRNA, it has been predicted that target mRNAs are stored in the complex of SGs under heat stress conditions through their association with UBP1b. In order to determine whether or not the target candidates were stabilized by UBP1b, an RNA decay analysis was performed using cordycepin, a transcriptional inhibitor. Once transcription is inhibited, the rate at which the level of mRNA decreases over time is considered to represent the rate of degradation. In response to cordycepin treatment, the mRNA encoding a DnaJ domain-containing protein (AT3G13310) and AtSAP3 remained more stable in *UBP1b*-ox plants than in *Venus* control plants (Figure 4B). The rate of degradation of candidate target mRNAs was slower in *UBP1b*-ox plants than in *Venus* control plants. The half-life of *AT3G13310* and *AtSAP3* mRNAs was 5.3 h and 114.9 h, respectively in *UBP1b*-ox plants, which was approximately 3.5 and 55 times longer than in the *Venus* control plants, respectively. These results implicate that the mRNAs of *AT3G13310* and *AtSAP3* are positively protected by UBP1b from degradation. In contrast, the rate of mRNA degradation of six genes (*AT1G72416, AT1G56300, HSFA4A, HSFA3, SSADH*, and *WRKY25*) that exhibited higher levels of expression in *UBP1b*-ox plants than in *Venus* control plants, was not significantly different in *UBP1b*-ox plants than in *Venus* control plants (data not shown).

## Discussion

SGs appear in plant cytosol in response to the perception of environmental stresses, such as heat (Weber et al., 2008). Detailed information on the function of SGs, as well as their components, however, have not been clearly elucidated. SGs are cytoplasmic foci that function in translational silencing (Anderson and Kedersha, 2002) and that sequester stress-inducible mRNA transcripts which may play a role in stress response and adaptation (Anderson and Kedersha, 2002, 2006; Sorenson and Bailey-Serres, 2014). UBP1b protein, a component of SGs, interacts with the 3′-UTR of mRNAs and protects them from exonucleolytic degradation (Lambermon et al., 2000). UBP1b plays a central role in the accumulation of SGs under stress conditions (Weber et al., 2008). In the present study, UBP1b was shown to change its cellular localization in response to heat stress from the nucleus to within SGs (Figure 1). Furthermore, UBP1b was demonstrated to play an integral role in plant heat-stress tolerance. Additionally, two target candidate mRNAs (*AT3G13310*; *AtSAP3*) were identified by microarray and RNA decay analyses and the expression of several heat stress tolerance-related genes, such as the heat shock transcription factors (*HSFA4A* and *HSFA3*) and *WRKY25*, were shown to be higher in *UBP1b*-ox plants than in *Venus* control plants.

CLSM microscopy revealed that UBP1b SGs are formed in response to heat stress (40°C) in *UBP1b*-ox lines (Figure 1A). SGs were detected in both roots and leaves of *UBP1b*-ox plants when they were exposed to 40°C (Figures 1A,B). These results are consistent with a previous report (Weber et al., 2008) and indicate that heat stress response experiments can be conducted at 40°C. In the current study, a 42°C treatment was used for the heat tolerance assay of *UBP1b*-ox and *ubp1b* mutant plants because 42°C caused death in a portion of the wild-type plants. On the other hand, 40°C induced the formation of UBP1b SG without killing the plants. Therefore, a 40°C treatment was used for characterizing the transcriptome of *UBP1b*-ox and *Venus* control plants subjected to heat stress.

Microarray and RNA decay analyses identified two candidate UBP1b target genes encoding a DnaJ domain-containing protein (AT3G13310) and a stress-associated protein (AtSAP3)/AT2g27580 (Table 1, Figures 3, 4), both of which are involved in heat stress tolerance. The expression of these genes was higher in *UBP1b*-ox than in *Venus* control plants subjected to 22°C, and were also heat-inducible when plants of both lines were exposed to 40°C. Importantly, the rate of RNA degradation of these genes was slower in *UBP1b*-ox plants than it was in the *Venus* control plants. DnaJ domain-containing proteins function in maintaining protein homeostasis under environmental stress conditions (heat etc.) by stimulating the ATPase activity of chaperone proteins, such as the 70-kilodalton heat shock proteins (Hsp70s) (Rajan and D'silva, 2009; Chiu et al., 2013). AtSAPs have been reported to play a role in plant heat stress tolerance (Vij and Tyagi, 2006; Dixit and Dhankher, 2011; Kim et al., 2015). Mutants of *AtSAP5* exhibit a heat stress-sensitive phenotype (Kim et al., 2015) and overexpression of *AtSAP10* enhances tolerance to high temperature stress (Dixit and Dhankher, 2011). The results of the mRNA decay assay conducted at 22°C in the present study indicated that UBP1b functions even at non-stress temperatures. Based on the results of the RNA decay experiments, it is plausible to suggest that UBP1b interacts with the 3′-UTR domain of target mRNAs (*AT3G13310* and *AtSAP3*) and inhibits or prevents them from being degraded.

Microarray and RT-qPCR analyses also identified a set of heat stress-inducible genes, including two DnaJ domain-containing proteins (*AT1G72416* and *AT1G56300*), two heat shock transcription factors (*HSFA4A* and *HSFA3*), and a *WRKY25* that exhibited higher levels of expression in *UBP1b*-ox plants (Figures 3, 4, Figure S2) than in *Venus* control plants. Heat shock factor A4A *(HSFA4A*) regulates a set of heat-, H_2_O_2_-, and salt-responsive genes (Pérez-Salamó et al., 2014). Overexpression of *HsfA3* results in the induction of many heat-inducible genes and increases thermotolerance, while *hsfa3* knockout mutants exhibit reduced thermotolerance (Yoshida et al., 2008). *HsfA3* is one of the most highly upregulated heat-inducible genes in transgenic plants constitutively overexpressing the active form of *DREB2A*, which exhibit increased thermotolerance (Sakuma et al., 2006). Knockout mutants of *succinic semialdehyde dehydrogenase* (*SSADH, AT1G79440*) are sensitive to heat stress (Bouché et al., 2003). *WRKY25*-overexpressing plants exhibit enhanced heat tolerance, while *wrky25* knockout mutants exhibit a thermosensitive phenotype relative to wild-type plants (Li et al., 2009). The higher level expression of these heat stress-inducible genes in *UBP1b*-ox plants may contribute to the increased thermotolerance of *UBP1b*-ox plants observed in the current study. The RNA decay assay, however, did not reveal a significant difference between the rate of degradation of these candidate mRNAs' in *UBP1b*-ox plants vs. the *Venus* control plants (data not shown), suggesting that these genes may be the indirect targets of UBP1b.

## Conclusions

The study of the regulation of mRNA stability in response to stress has emerged as a new topic of research. In the present study, UBP1b was demonstrated to be a component of the machinery that controls the post-transcriptional regulation of gene expression as part of a mechanism to promote the survival of plants subjected to heat stress. Under heat stress conditions, the formation of UBP1b SG complexes is induced in both roots and shoots. Phenotypic analysis of *UBP1b*-ox and *ubp1b* mutant plants revealed that UBP1b plays an integral role in plant heat stress tolerance. Microarray analysis identified 117 heat stress-inducible genes whose expression was higher in *UBP1b*-ox plants than in control plants. Two candidate mRNA targets of UBP1b (a DnaJ heat shock protein, *AT3G13310* and a stress-associated protein, *AtSAP3*) that are involved in heat stress response and tolerance are highly expressed and maintain stability under the effect of UBP1b. The identification of *UBP1b* target genes and the verification of UBP1b-interacting proteins will provide a more comprehensive understanding of how mRNAs are regulated when plants experience environmental stress. Furthermore, an understanding of the regulatory mechanisms governing mRNA stability during abiotic stress may help to enable the development of stress-tolerant plants.

## Author contributions

CCN, KN, AM, and MS designed the study, CCN, KN, AM, SK, YK, KT, and MT performed the research. CCN, KN, and AM analyzed the data, CCN, KN, AM, and MS discussed the data and wrote the manuscript.

### Conflict of interest statement

The authors declare that the research was conducted in the absence of any commercial or financial relationships that could be construed as a potential conflict of interest.
