# Supplementary material for: Oligouridylate Binding Protein 1b Plays an Integral Role in Plant Heat Stress Tolerance
Source: Front Plant Sci. 2016 Jun 17;7:853. doi: 10.3389/fpls.2016.00853 (PMC4911357; doi:10.3389/fpls.2016.00853)
Supplement: Supplementary file 2 [file Image1.pdf]

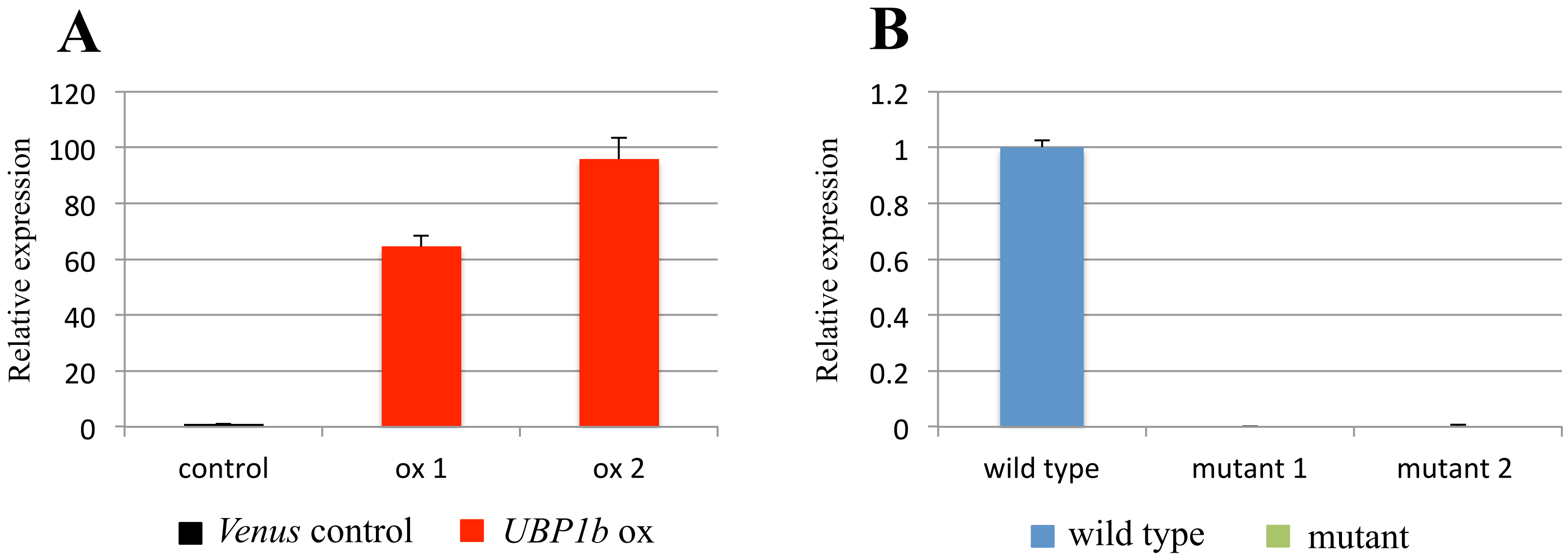

Figure S1. RT-qPCR analysis of *UBP1b* gene in *UBP1b*-ox, *ubp1b* mutants and *Venus* control plants under non-stress conditions. A. RT-qPCR analysis of *UBP1b* gene in *UBP1b*-ox and *Venus* control plants. B. RT-qPCR analysis of *UBP1b* gene in *ubp1b* mutants and WS wild type plants. Relative expression levels of *UBP1b* gene is shown. Data represent the mean ± sd of three biological replicates.
