# Supplementary material for: Oligouridylate Binding Protein 1b Plays an Integral Role in Plant Heat Stress Tolerance
Source: Front Plant Sci. 2016 Jun 17;7:853. doi: 10.3389/fpls.2016.00853 (PMC4911357; doi:10.3389/fpls.2016.00853)
Supplement: Supplementary file 3 [file Image2.pdf]

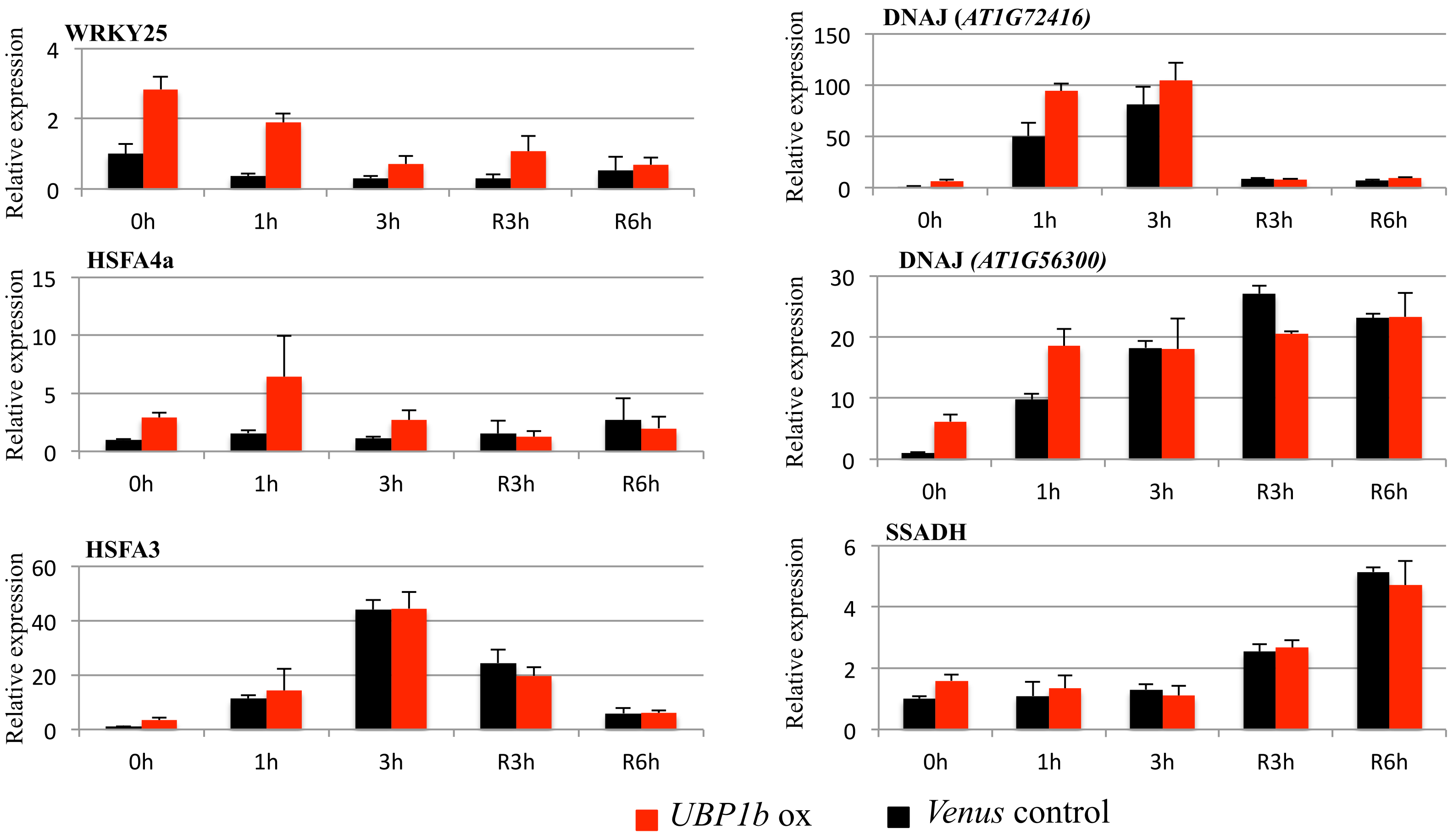


**Figure S2**. RT-qPCR analysis of target candidates of UBP1b in *UBP1b*-ox and *Venus* control plants.
The following genes exhibited higher levels of relative expression in *UBP1b*-ox plants exposed to a heat stress (40º C for 1 or 3 h) than in *Venus* control plants as determined by RT-qPCR: *WRKY25 (AT2G30250), DNAJ (AT1G72416), HSFA4A (AT4G18880), DNAJ (AT1G56300), HSFA3(AT5G03720)*, *succinic semialdehyde dehydrogenase* (*SSADH*, *AT1G79440*). RT-qPCR analyses of *UBP1b*-ox and *Venus* control plants were conducted on plants exposed to non-stress (22º C) conditions and recovery conditions (3 h and 6 h after transfer back to 22º C). *y*-axis: Relative expression levels of the target candidate genes relative to their expression in *Venus* control plants exposed to non-stress conditions.
